# Corn and Husks

**Published:** 1891-09-19

**Authors:** 


### THE DOCTOR'S ARMCHAIR.

CORN AND HUSKS.

PROFESSOR MAX MULLER, who presided over the Anthropological section at the Meeting of the British Association this year, devoted a great part of his presidential address to the reproving of youthful scientists of the omniscient and infallible type. Those of us who have passed through the classes of chemistry, botany, zoology, physiology, and the other branches of natural science which help to form the medical curriculum, can call to mind the profound veneration we used to feel for the vast knowledge and erudition of the learned professors who strove to teach us the elements of those sciences, and the still deeper admiration which filled our minds, and shone out of our eyes as we contemplated the greater knowledge, and the far more unquestionable infallibility and authority of the wonderful young honours graduates, who condescended to act as their professional assistants. It was one of the delightful delusions of those early days that our particular medical school was beyond question the greatest, most famous, and most learned of all medical schools; and that the present professors, but more especially their class assistants, were nothing short of miracles of the widest and most profound learning in their own and every other branch of natural science.

Alas! we know better now. The medallist of our senior class no longer assumes the proportions of a hero; the class assistant, even with his spectacles and his frock coat, thrills us with admiration no more; worst of all, the grave and grey-bearded professor who was the unquestioned and unquestionable pope of our scientific church, is known now to have been but an ordinary man—so very ordinary indeed that we feel some of the withering "self-contempt" which Tennyson so kindly prescribed for his cousin, as we think sadly of the pompous little man's tones of tremendous authority, and of his emptiness of scientific worth.

The venerable Max Müller is very severe upon those persons who are known as "Armchair Scientists." They conduct their original researches in the depths of the savage jungles of their libraries; and face unheard of dangers among the obscure mountain fastnesses of their topmost shelves. These are the gentlemen who know all about it. They can settle the question of the origin of man before you can take half a dozen whiffs at your cigarette. The ancient religions of the world have no mysteries for them. Totemism is the great word to conjure with. Every race and tribe had its own Totem. The fact is as plain as a pike-staff, and explains everything. What more would you have?

Mr. Max Müller demands a good deal more. "I do not mean to depreciate the labours of so-called *dilettanti*," he says. "After all, *dilettanti* are lovers of knowledge." Of course they are, exactly as ladies are lovers of fine clothes, and for the same reason. A man who has no claim to distinction of any kind thinks he establishes a claim by reading up the theories of half a dozen more or less ignorant scientific dabblers, and setting up a seventh theory on his own private account; which theory, when it has been set up, is merely a hotch-potch of all the other theories, seasoned with a suspicion of his own special ignorance. Thus fearfully and wonderfully dressed, he proclaims the conviction, *urbi et orbi*, that no scientific Solomon in all his glory was ever arrayed so resplendently as he.

"You know," says our learned Professor, "how widely classical scholars differ on the character of Greeks and Romans, on the meaning of their customs, the purpose of their religious ceremonies—nay, the very essence of their gods. And yet there was a time, not very long ago, when anthropologists would rely on the descriptions of casual travellers, who, after spending a few weeks, or even a few years, among tribes whose language was utterly unknown to them, gave the most marvellous accounts of their customs, their laws, and even of their religion. It may be said that anybody can describe what he sees, even though unable to converse with the people. I say, decidedly, No." Says Mr. Fison, late missionary in Fiji: "When a European has been living for two or three years among savages, he is fully convinced that he knows all about them: when he has been ten years or so amongst them, if he be an observant man, he knows that he knows very little about them, and so he begins to learn." Quite so! The gaseous anthropologist is being found out exactly as the alchemist, the jerry builder, and all sorts of ancient and modern prestidigitateurs have been, and will continue to be found out. Mr. Andrew Lang, in his easy and pleasant contributions to a well-known morning paper, has pricked a good many windbags of extremely imposing appearance. The present writer has a near relation who has been for ten years a missionary in one of the so-called savage parts of Africa. It makes this good missionary both amused and angry when casual travellers, who show no special marks of genius as they sit at the hospitable mission table, return to England after a six or eight weeks' tour, and write books full of high-sounding phrases about the aborigines and their ways. As well might the aborigines write books about their European visitors, says the experienced missionary.

Simple folks are beginning to take a much juster measure of ambitious pretenders to science than they did a score of years ago. One finds that "missing links" cannot be brought to light, that men with rudimentary tails are nowhere discoverable, that religion has not been banished from the world, and that the "superstition" about eternal justice and truth still holds its ground, even among cultured and intelligent people. "The time will come," says Max Müller, "when no anthropologist will venture to write on anything concerning the inner life of man without having himself acquired a knowledge of the language in which that inner life finds its truest expression. . . . You have only to look, for instance, at the description given of the customs, the laws, the legends, and the religious convictions of the people of India about a hundred years ago, and before Sanscrit began to be studied, and you will be amazed at the utter caricature that is often given there of the intellectual state of the Brahmans compared with what we know of it now from their own literature."

The scientific phrase-makers have had a long innings. They have not ruled the world with words and formulæ so long as the metaphysicians and theologians ruled it, but they have ruled it quite long enough. The plain man, "the mere justice-loving man," as Carlyle called him, is now entitled to his turn. He is beginning to know genuine from counterfeit scientific coin when he sees it. He is angry that he has accepted so many base half-crowns in the past. He will not repeat his folly, at least with his eyes open. The man of real science, the Faraday, the Newton, the Edison, shall have higher exaltation than ever; but the thief who steals other men's discoveries and ideas, and, with some wretched little modification, proclaims them as his own, shall be vigorously kicked out into the limbo appointed for those basest of all cheats who live by the passing of spurious coin. Professor Max Müller has deserved well of all genuine scientific workers in pouring out the vials of his good-natured contempt upon the wind-bags. It is hoped they will take it to heart. They may depend upon it their innings is played out, whether they know it or not. Better a single bushel of whole-some scientific corn, of which bread can be made, than whole shiploads of mere chaff which does not hold enough of sound grain for the feeding of one spring chicken.

